# New Alkaloids from *Aconitum stapfianum*

**DOI:** 10.1007/s13659-015-0075-1

**Published:** 2015-10-12

**Authors:** Tian-Peng Yin, Le Cai, Ying Li, Yun-Shan Fang, Li Peng, Zhong-Tao Ding

**Affiliations:** Key Laboratory of Medicinal Chemistry for Nature Resource, Ministry of Education, School of Chemical Science and Technology, Yunnan University, Kunming, 650091 China

**Keywords:** *Aconitum stapfianum*, Ranunculaceae, Diterpenoid alkaloid, Benzamide, Stapfianine

## Abstract

**Abstract:**

Nineteen alkaloids, including a new C_19_-diterpenoid alkaloid stapfianine A (**1**) and a new benzamide derivative stapfianine B (**2**) were isolated from the roots of *Aconitumstapfianum*. Their structures were established on the basis of extensive spectroscopic analyses (IR, HRESIMS, 1D and 2D NMR).

**Graphical Abstract:**

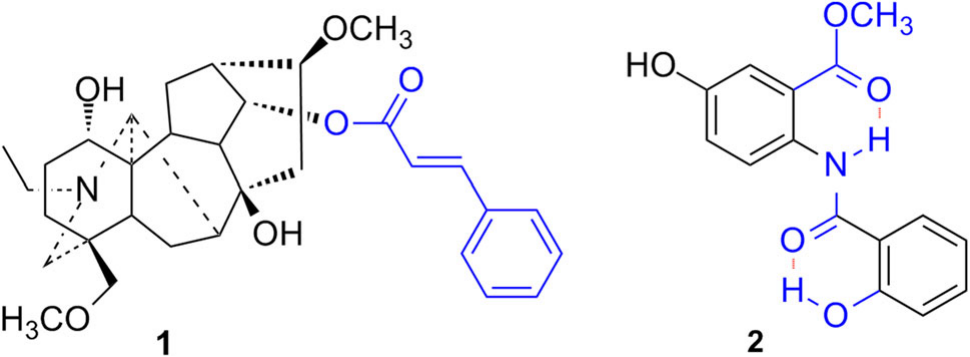

**Electronic supplementary material:**

The online version of this article (doi:10.1007/s13659-015-0075-1) contains supplementary material, which is available to authorized users.

## Introduction

**Fig. 1:**
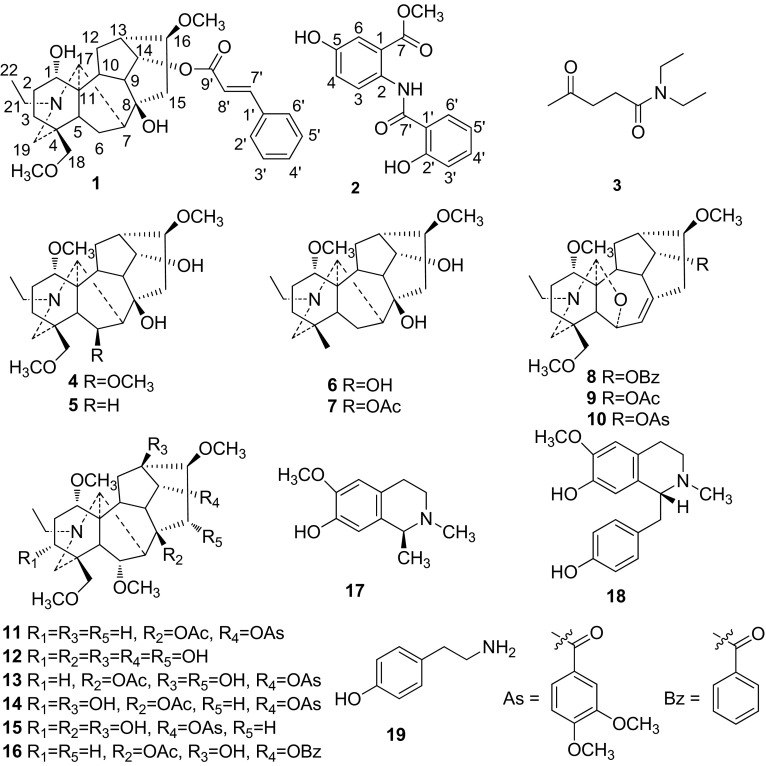
Structures of alkaloids isolated from *Aconitum stapfianum*

*Aconitumstapfianum* Hand.-Mazz. belongs to the genus *Aconitum* of the Ranunculaceae, and is distributed mainly at an altitude of 2800–3400 m in the northwest of Yunnan Province in China [1]. Up to now, only four diterpenoid alkaloids have been isolated from *A. stapfianum* [2]. As part of our continuous work on the discovery of bioactive ingredients from the *Aconitum* plants [3, 4], an phytochemical investigation on the roots of *A. stapfianum* was carried out to afford nineteen alkaloids (Fig. 1), including a new C_19_-diterpenoid alkaloid stapfianine A (**1**), a new benzamide derivative stapfianine B (**2**), and a known amide 4-oxo-pentanoic acid dimethylamide (**3**) found in nature for the first time. Their structures were established on the basis of extensive spectroscopic analyses. In this paper, the isolation and structure determination of these alkaloids are described.

## Results and Discussion

**Fig. 2:**
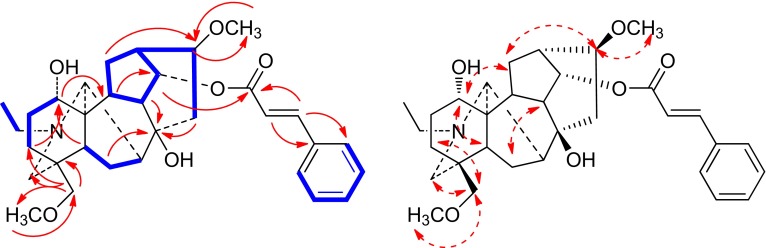
Key ^1^H-^1^H COSY (), HMBC () and ROESY () correlations of compound **1**

Compound **1** was isolated as a white amorphous powder and its molecular formula was deduced to be C_32_H_43_NO_6_ by HRESIMS at *m/z* 538.3169 [M+H]^+^. The NMR spectra of **1** showed the presence of a ester carbonyl (*δ*_C_ 166.6 s), a characteristic disubstituted double bond (*δ*_H_ 6.41, d, *J* = 16.0 Hz, 7.65, d, *J* = 16.0 Hz; *δ*_C_ 118.0 d, 145.4 d) and a mono-substituted benzene (*δ*_H_ 7.36 m, 7.36 m, 7.50 m; *δ*_C_ 128.3 d, 129.0 d, 130.5 d, 134.4 s), which were assigned to a cinnamoyl group [5]. Additionally, an N-ethyl group (*δ*_H_ 1.10, t, *J* = 7.2 Hz; *δ*_C_ 13.1 q, 48.6 t) and two methoxyl groups were identified in the NMR spectra as well. Compound **1** possesses 21 carbons except for the cinnamoyl and methoxyl groups, in combination with biogenetic consideration, suggest that **1** might be an aconitine-type C_19_-diterpenoid alkaloid [6]. The cinnamoyl group was placed at C-14 according to the HMBC correlation from H-14 (*δ*_H_ 5.01, t, *J* = 4.8 Hz) to C-9′ (*δ*_C_ 166.6 s) (Fig. 2), while the *α*-orientation of the cinnamoyl group was confirmed by the ROESY correlation between H-10 and H-14. Two methoxyl groups were placed at C-16 and C-18 on the basis of the HMBC correlations from OCH_3_-16 (*δ*_H_ 3.26, s) to C-16 (*δ*_C_ 82.3 d), from OCH_3_-18 (*δ*_H_ 3.30, s) to C-18 (*δ*_C_ 79.2 t), respectively. In addition, the ROESY correlations between H-13 and OCH_3_-16 demonstrated the *β*-orientation of OCH_3_-16. A hydroxyl group should be located at C-8 according to the HMBC correlations from H-15, H-6 and H-9 to C-8. Additionally, a signal at *δ*_H_ 3.74 was attributed to H-1*β*, suggesting the presence of an OH-1*α* [7, 8], which was further supported by the ROESY correlation between H-1 and H-5. Therefore, the structure of compound **1** was determined as stapfianine A, with its assigned NMR data listed in Table 1.

**Table 1:** NMR spectroscopic data (400 MHz for ^1^H and 100 MHz for ^13^C, CDCl_3_) for compound **1**

No.	*δ*_H_ *J* (Hz)	*δ*_C_	No.	*δ*_H_ *J* (Hz)	*δ*_C_
1	3.74 brs	72.2 d	16	3.31 m	82.3 d
2	1.59 m	27.8 t	17	2.75 brs	63.8 d
	1.59 m				
3	1.62 m	26.7 t	18	3.13 ABq (7.4)	79.2 t
	1.88 m			2.99 ABq (8.8)	
4		37.3 s	19	2.32 ABq (11.2)	56.6 t
				2.04 ABq (11.2)	
5	1.93 m	43.5 d	21	2.50 m	48.6 t
				2.44 m	
6	1.84 m	25.2 t	22	1.10 t (7.2)	13.1 q
	1.63 m				
7	2.03 brs	45.8 d	OCH_3_-16	3.26 s	56.2 q
8		75.0 s	OCH_3_-18	3.30 s	59.5 q
9	2.30 m	44.8 d	1′		134.4 s
10	1.94 m	43.6 d	2′, 6′	7.50 m	128.3 d
11		49.0 s	3′, 5′	7.36 m	129.0 d
12	2.11 m	29.3 t	4′	7.36 m	130.5 d
	1.73 m				
13	2.63 m	37.4 d	7′	7.65 d (16.0)	145.4 d
14	5.01 t (4.8)	77.2 d	8′	6.41 d (16.0)	118.0 d
15	2.34 m	42.6 t	9′		166.6 s
	2.03 m				

**Fig. 3:**
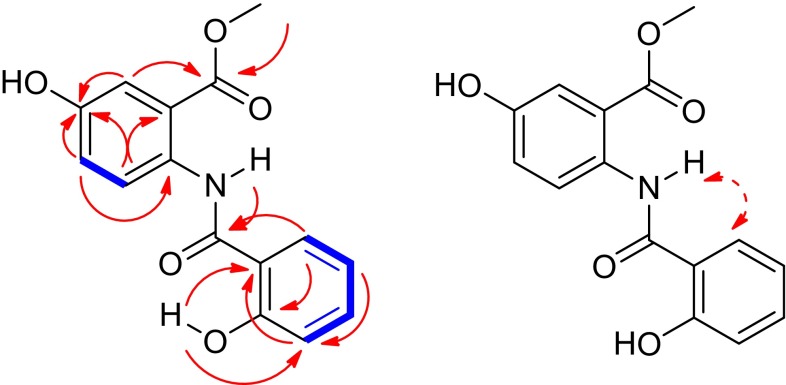
Key ^1^H-^1^H COSY (), HMBC () and ROESY () correlations of compound **2**

Compound **2** was isolated as a white amorphous powder and its molecular formula was deduced to be C_15_H_13_NO_5_ with an unsaturation degree of ten by HRESIMS at *m/z* 310.0673 [M+Na]^+^. The ^1^H-NMR spectrum of **2** showed signals of a methoxyl group (*δ*_H_ 3.95, s), a 1,2,4-trisubstituted aromatic ring (*δ*_H_ 7.11, dd, *J* = 8.8 Hz, 2.4 Hz; 7.54, d, *J* = 2.4 Hz; 8.63, d, *J* = 8.8 Hz), and a 1,2-disubstituted aromatic ring (*δ*_H_ 7.01, d, *J* = 8.4 Hz; 7.43, t, *J* = 7.6 Hz; 6.96, t, *J* = 7.6 Hz; 7.76, d, *J* = 8.0 Hz) (Table 2). The ^13^C-NMR spectrum revealed 15 carbons resonances, corresponding to the above protonated units and two carbonyl groups (*δ*_C_ 168.8 s, 168.9 s). The data summarized above, in combination with biogenetic consideration, suggested that compound **2** might be a benzamide derivatives [9, 10]. A methyl ester group was placed at C-2 on the basis of the HMBC correlations from OCH_3_-7, H-3 to C-7 (*δ*_C_ 168.9 s), and H-6 to C-2 (Fig. 3). Two hydroxyl groups could be located at C-4 and C-2′ on the basis of the HMBC correlations from H-6, H-3 to C-4, and OH-2′ to C-2′, respectively. A quaternary carbon (*δ*_C_ 134.4 s) *ortho* to the methyl ester was assigned to connect with the N-atom of the amide group, which caused strong hydrogen bonding between –NH (*δ*_H_ 11.94, s) and the carbonyl of the ester [11]. Similarly, the downfield shift of OH-2 (*δ*_H_ 12.34, s) caused by intramolecular hydrogen bonding between the amide carbonyl and OH-2′ suggested that the amide group connected with C-1′, which was further supported by the HMBC correlation from H-6′ to C-7′ and the ROESY correlation between H-6 and –NH [12]. Therefore, the structure of compound **2** was determined as stapfianine B, with its assigned NMR spectroscopic data listed in Table 2.

**Table 2:** NMR spectroscopic data (400 MHz for ^1^H and 100 MHz for ^13^C, CDCl_3_) for compound **2**

No	*δ*_H_ *J* (Hz)	*δ*_C_	No	*δ*_H_ *J* (Hz)	*δ*_C_
1		134.4 s	1′		115.3 s
2		117.1 s	2′		162.2 s
3	7.54 d (2.4)	117.2 d	3′	7.01 d (8.4)	118.8 d
4		151.4 s	4′	7.43 t (7.6)	134.6 d
5	7.11 dd (8.8, 2.4)	122.2 d	5′	6.96 t (7.6)	119.4 d
6	8.63 d (8.8)	122.7 d	6′	7.76 d (8.0)	126.2 d
7		168.9 s	7′		168.8 s
OCH_3_-7	3.95 s	52.9 q	NH	11.94 s	
			OH-2′	12.34 s

Based on spectroscopic analyses and comparison with the literature, the known alkaloids were identified as 4-oxo-pentanoic acid dimethylamide (**3**) [13], 6-epichasmanine (**4**) [14], talatisamine (**5**), sachaconitine (**6**), 14-acetylsachaconitine (**7**) [15], franchetine (**8**), vilmorrisine (**9**) [16], kongboendine (**10**) [17], vilmorrianine C (**11**), aconine (**12**) [18], crassicauline A (**13**) [19], yunaconitine (**14**), 8-deacetylyunaconnitine (**15**) [15], chasmaconitine (**16**), *N*-methylisosalsoline (**17**) [20], (−)-*N*-methylcoclaurine (**18**) [21] and tyramine (**19**) [22]. Compound **3** was isolated for the first time from a natural source in this study. Besides, compounds **4**, **6**–**12**, **15**–**19** were isolated from this species for the first time.

## Experimental Section

### General Experimental Procedures

Optical rotation was measured with a Jasco P-1020 digital polarimeter (JASCO, Tokyo, Japan). A Shimadzu UV–Vis 2550 spectrometer (Shimadzu, Kyoto, Japan) was used for collection of UV spectra. NMR spectra were acquired with a Bruker AM-400 spectrometer (Bruker, Karlsruhe, Germany) using TMS as the internal reference. A Nicolet Magna-IR 550 spectrometer (Thermo Nicolet, Madison, USA) was used for scanning IR spectroscopy with KBr pellets. Melting points were determined on a XRC-1 Melting Point Apparatus (Sichuan University Science Instrument, Chengdu, China) and were not corrected. ESI–MS analyses were recorded with an Agilent G3250AA (Agilent, Santa Clara, USA) and Auto Spec Premier P776 spectrometer (Waters, Milford, USA). Silica gel (200–300 mesh and 300–400 mesh; Qingdao Marine, Qingdao, China) and Sephadex LH-20 (GE Healthcare, Fairfield, USA) were used for column chromatography (CC). GF254 plates (Qingdao Marine, Qingdao, China) were used for thin layer chromatography, and spots were visualized by spraying with modified Dragendorff’s reagent or 10 % H_2_SO_4_ in ethanol followed by heating.

### Plant Material

Roots of *A.* *stapfianum* were collected from Dali Bai Autonomous Prefecture of Yunnan Province in China in December 2012, and identified by professor Shu-Gang Lu from School of Life Sciences, Yunnan University. A voucher specimen (2012-yc-2) is deposited in the Key Laboratory of Medicinal Chemistry for Natural Resource, Ministry of Education, and Kunming, China.

### Extraction and Isolation

Air-dried and powdered roots (8.0 kg) of *A.* *stapfianum* were percolated with 0.5 % HCl. The aqueous acidic solution was basified with ammonia (10 %) to pH 9.0 and then extracted with EtOAc. Removal of the solvent under reduced pressure afforded the total crude alkaloids (85 g) as yellowish amorphous powder.

The total alkaloids were subjected to silica gel CC eluted with CHCl_3_–CH_3_OH gradient system (100:1 to 1:1) to give nine fractions (FrA–FrI). FrA (38.0 g) was further subjected to silica gel CC [petroleum ether (PE)–acetone–diethylamine, 100:5:1 to 100:20:1] to give five fractions (FrA1–FrA5). Further silica gel CC purification of FrA1 (0.7 g) was accomplished by elution with PE–acetone–diethylamine, (100:5:1 to 100:10:1) to afford compounds **6** (2.0 mg) and **7** (2.0 mg). FrA2 (4.7 g) was subjected to silica gel CC (PE–acetone–diethylamine, 100:10:1) to yield **1** (12.0 mg), **4** (37.0 mg) and **5** (3.5 g). FrA3 (0.9 g) was subjected to silica gel CC (CHCl_3_–CH_3_OH, 20:1) to yield compounds **11** (12.0 mg) and **13** (132.0 mg). FrA5 (27.0 g) was subjected to silica gel CC (PE–acetone–diethylamine, 100:5:1 to 100:20:1) to yield compounds **14** (23.0 g). Further silica gel CC purification of FrC (4.2 g) was accomplished by elution with PE–acetone–diethylamine, (100:10:1 to 100:20:1) to afford **15** (3.5 g), **16** (3.0 mg) and **17** (36.5 mg). FrE (1.9 g) was subjected to silica gel CC (CHCl_3_–CH_3_OH, 30:1 to 5:1) to yield compounds **2** (14.0 mg) and **3** (13.5 mg). FrF (2.7 g) was subjected to silica gel CC (PE–acetone–diethylamine, 100:5:1) to yield compounds **8** (2.0 mg), **9** (3.5 mg), and **10** (2.5 mg). FrG (0.7 g) was subjected to silica gel CC (PE–acetone–diethylamine, 100:5:1 to 100:10:1) to yield compounds **18** (8.5 mg), **19** (2.5 mg), and **12** (25.0 mg).

### Stapfianine A (**1**)

White amorphous powder; m.p. 76–77 °C, [*α*]$_{\text{D}}^{ 2 0}$ +4.19 (*c* 2.5, CH_3_OH), IR (KBr, cm^−1^): ν_max_ 3437, 2927, 2354, 1714, 1633, 1452, 1175, 1101. For ^1^H- and ^13^C-NMR spectroscopic data, see Table 1. HRESIMS *m/z*: 538.3169 [M+H]^+^ (calcd for C_32_H_44_NO_6_, 538.3163).

### Stapfianine B (**2**)

Yellow amorphous powder; m.p. 128–130 °C; IR (KBr, cm^−1^): ν_max_ 3420, 1695, 1645, 1612, 1524, 1444, 1230, 1069, 979. For ^1^H- and ^13^C-NMR spectroscopic data, see Table 2. HRESIMS *m/z*: 310.0673 [M + Na]^+^ (calcd for C_15_H_13_NO_5_Na, 310.0691).

## Electronic supplementary material

Supplementary material 1 (DOCX 1721 kb)
