# Supplementary material for: New Alkaloids from Aconitum stapfianum
Source: Nat Prod Bioprospect. 2015 Oct 12;5(6):271–5. doi: 10.1007/s13659-015-0075-1 (PMC4681710; doi:10.1007/s13659-015-0075-1)
Supplement: Supplementary file 1 — Supplementary material 1 (DOCX 1721 kb) [file 13659_2015_75_MOESM1_ESM.docx]

**Electronic Supporting Information**

**New Alkaloids from *Aconitum stapfianum***

Tian-Peng Yin · Le Cai · Ying Li · Yun-Shan Fang· Li Peng· Zhong-Tao Ding

Tian-Peng Yin and Le Cai have contributed equally to this work.

T.P. Yin · L. Cai · Y. Li · Y.S. Fang· L. Peng · Z.T. Ding (🖂)

Key Laboratory of Medicinal Chemistry for Nature Resource, Ministry of Education, School of Chemical Science and Technology, Yunnan University. Kunming 650091, China

e-mail: [ztding@ynu.edu.cn](mailto:ztding@ynu.edu.cn) (Z.T. Ding).

**Contents**

**Fig S1**. ^1^H NMR spectrum of **1** in CDCl_3_.

**Fig S2**. ^13^C NMR spectrum of **1** in CDCl_3_.

**Fig S3**. ^1^H-^1^H COSY spectrum of **1** in CDCl_3_.

**Fig S4**. HSQC spectrum of **1** in CDCl_3_.

**Fig S5**. HMBC spectrum of **1** in CDCl_3_.

**Fig S6**. ROESY spectrum of **1** in CDCl_3_.

**Fig S7.** HR-ESI-MS spectrum of **1**.

**Fig S8**. ^1^H NMR spectrum of **2** in CDCl_3_.

**Fig S9**. ^13^C NMR spectrum of **2** in CDCl_3_.

**Fig S10**. ^1^H-^1^H COSY spectrum of **2** in CDCl_3_.

**Fig S11**. HSQC spectrum of **2** in CDCl_3_.

**Fig S12**. HMBC spectrum of **2** in CDCl_3_.

**Fig S13**. ROESY spectrum of **2** in CDCl_3_.

**Fig S14.** HR-ESI-MS spectrum of **2**.


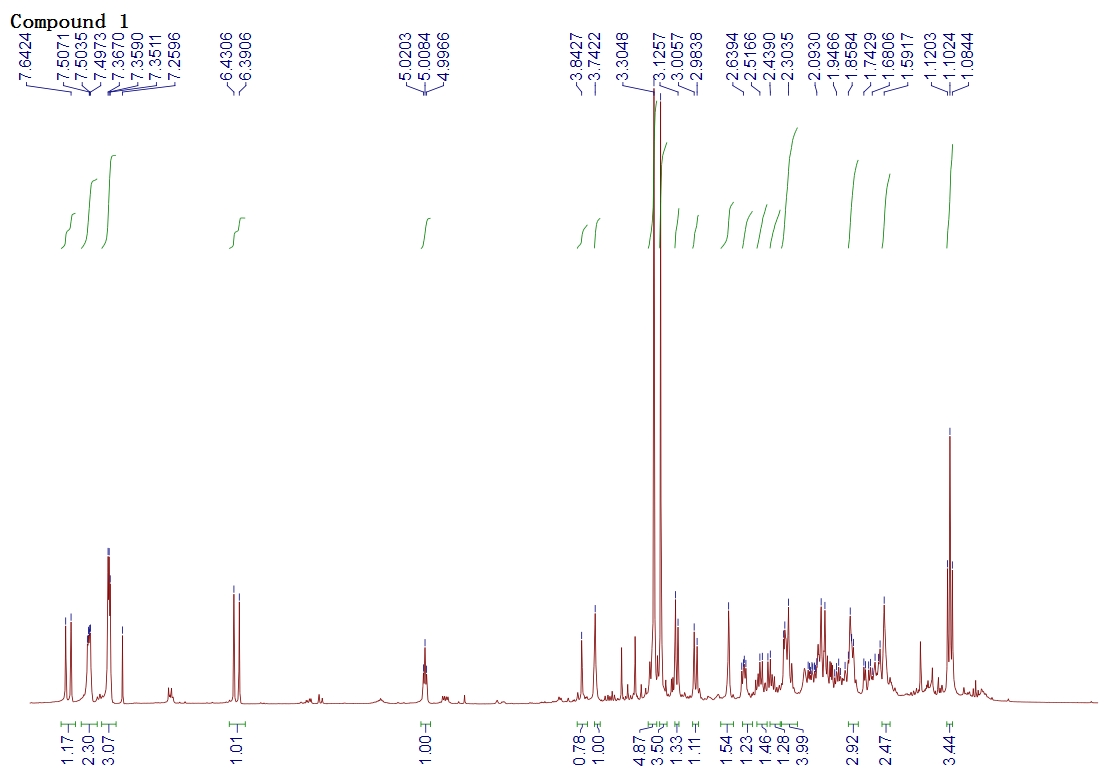


**Fig S1**. ^1^H NMR spectrum of **1** in CDCl_3_.


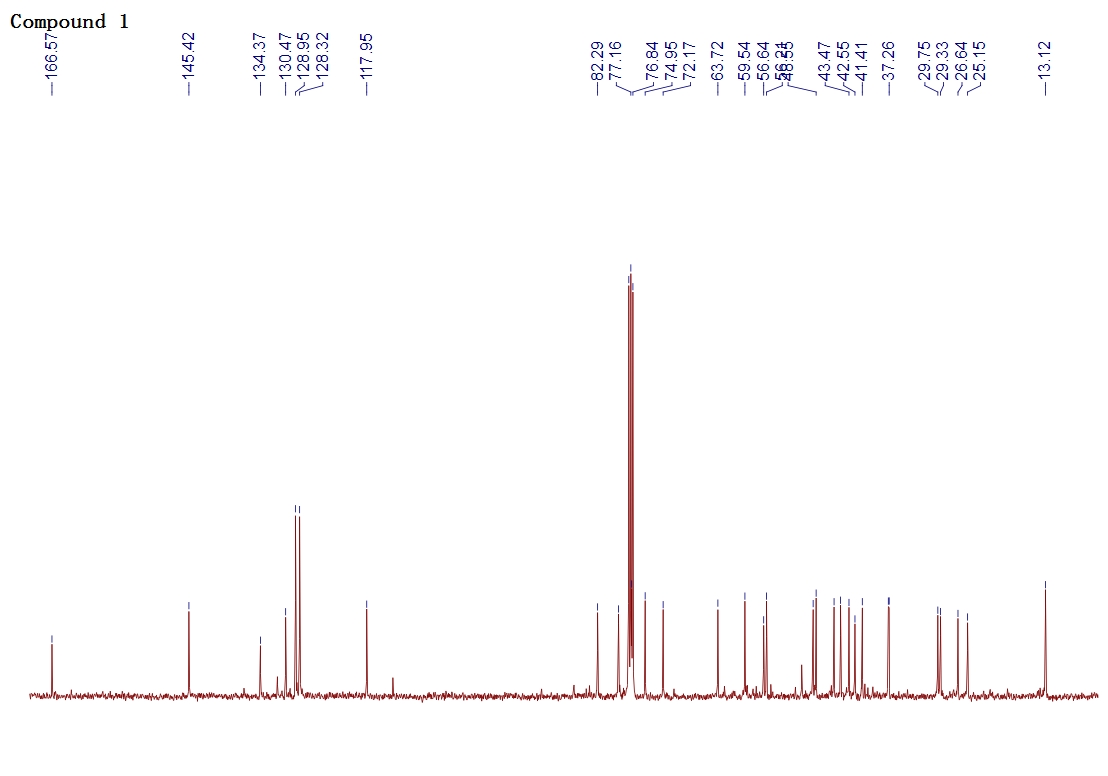


**Fig S2**. ^13^C NMR spectrum of **1** in CDCl_3_.


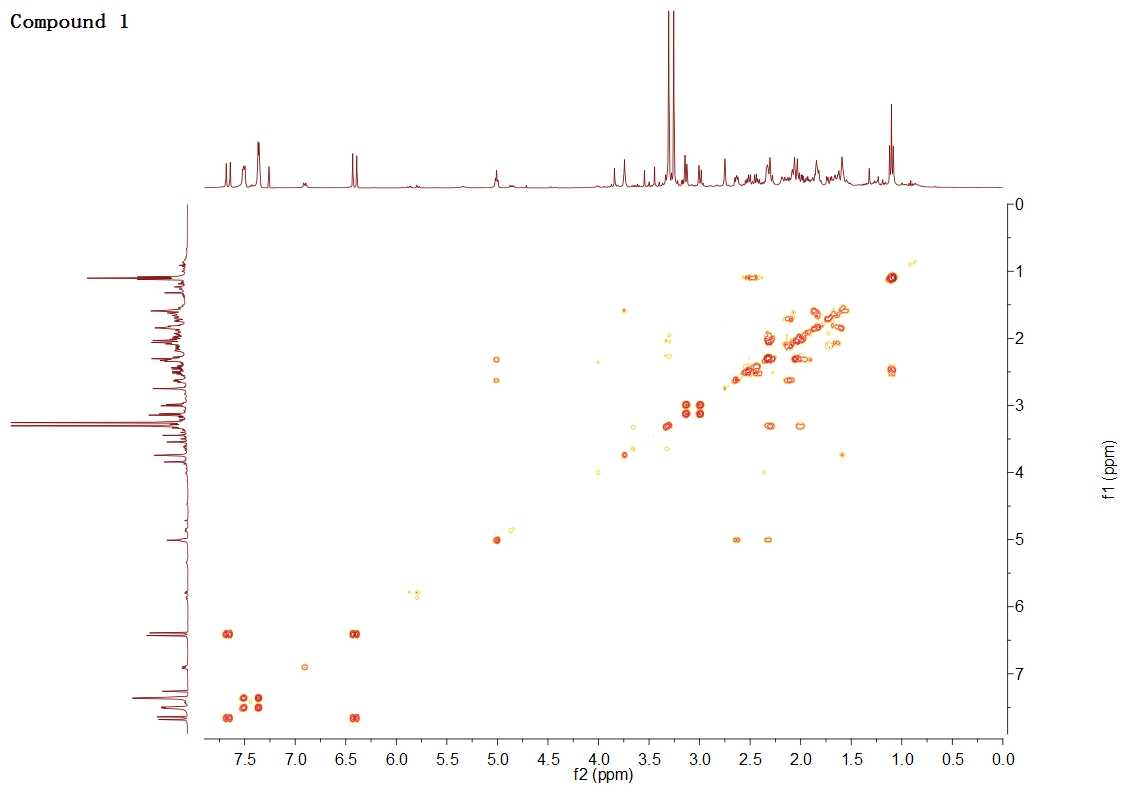


**Fig S3**. ^1^H-^1^H COSY spectrum of **1** in CDCl_3_.


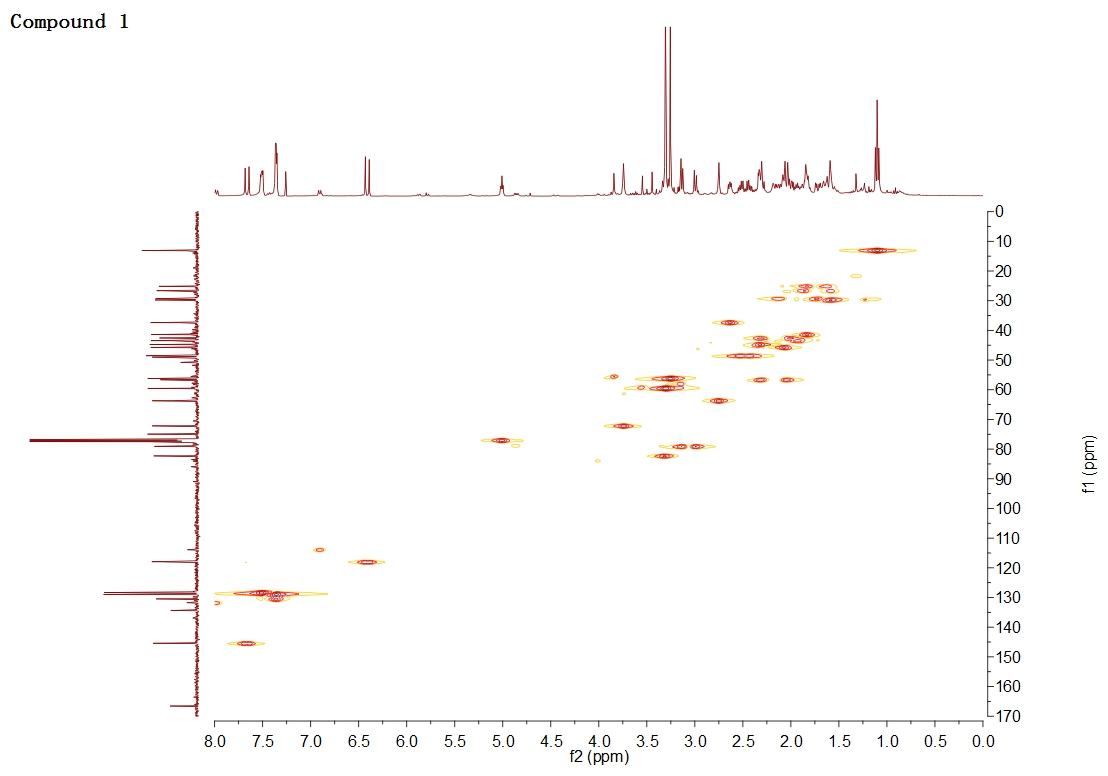


**Fig S4**. HSQC spectrum of **1** in CDCl_3_.


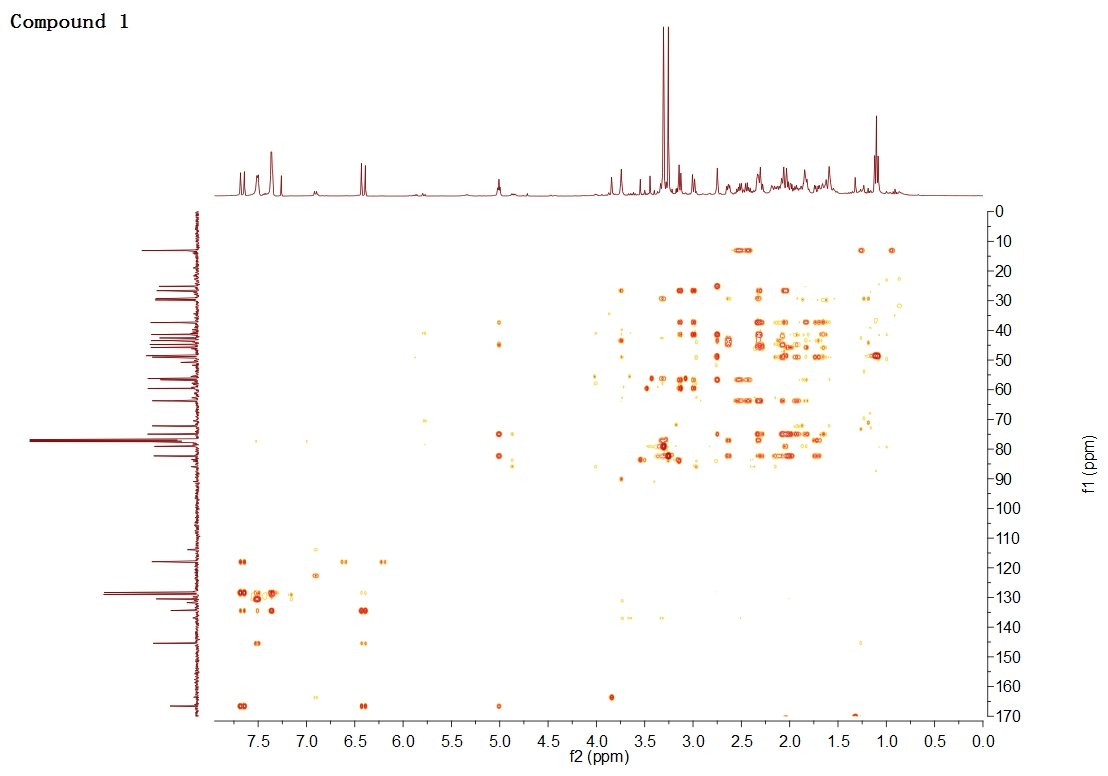


**Fig S5**. HMBC spectrum of **1** in CDCl_3_.


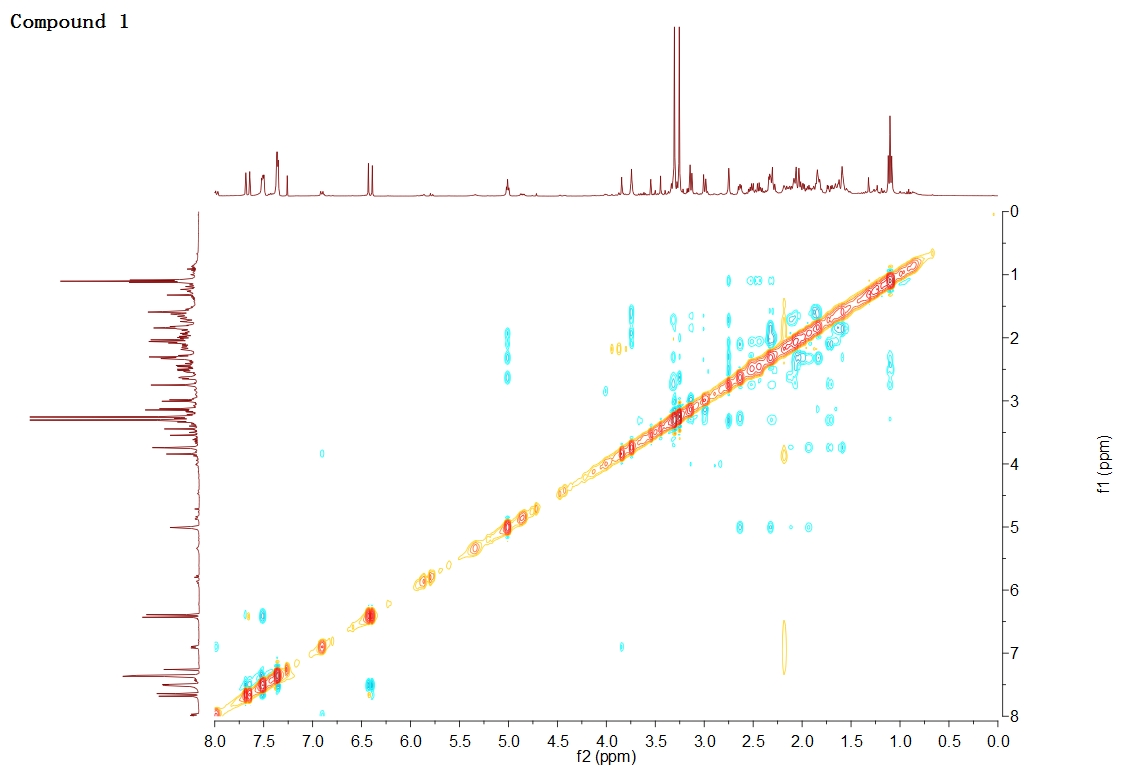


**Fig S6**. ROESY spectrum of **1** in CDCl_3_.

**Fig S7**. HR-ESI-MS spectrum of **1**.

**
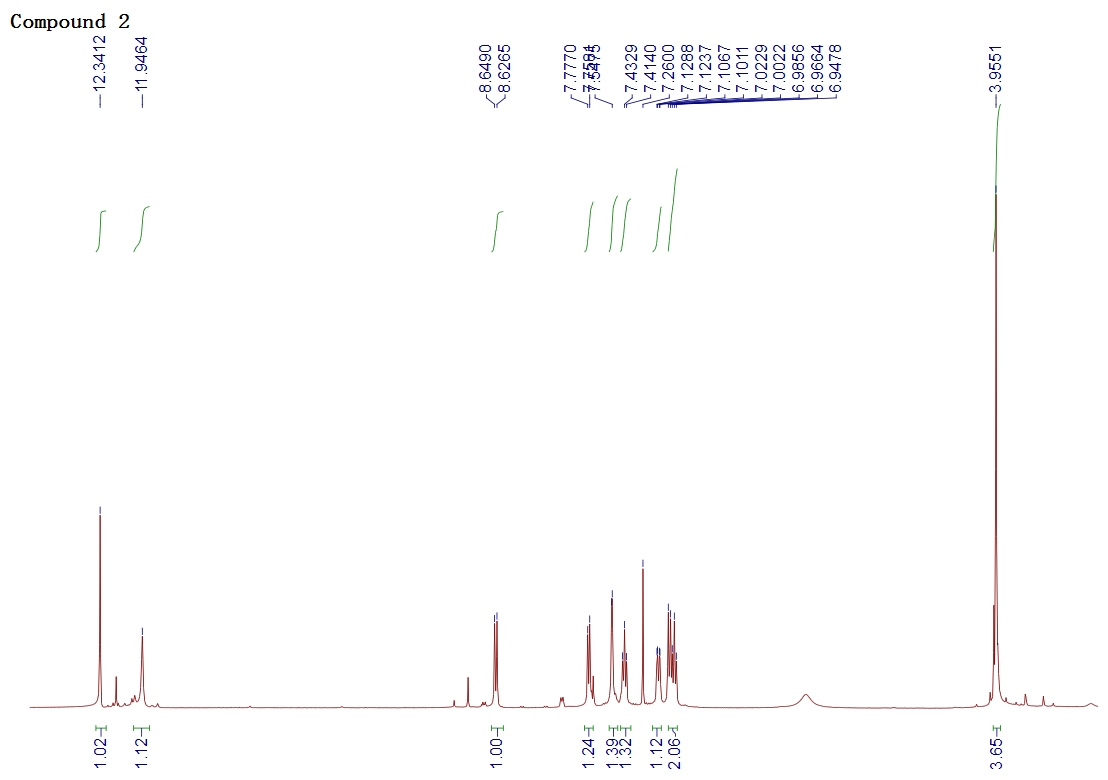
**

**Fig S8**. ^1^H NMR spectrum of **2** in CDCl_3_.


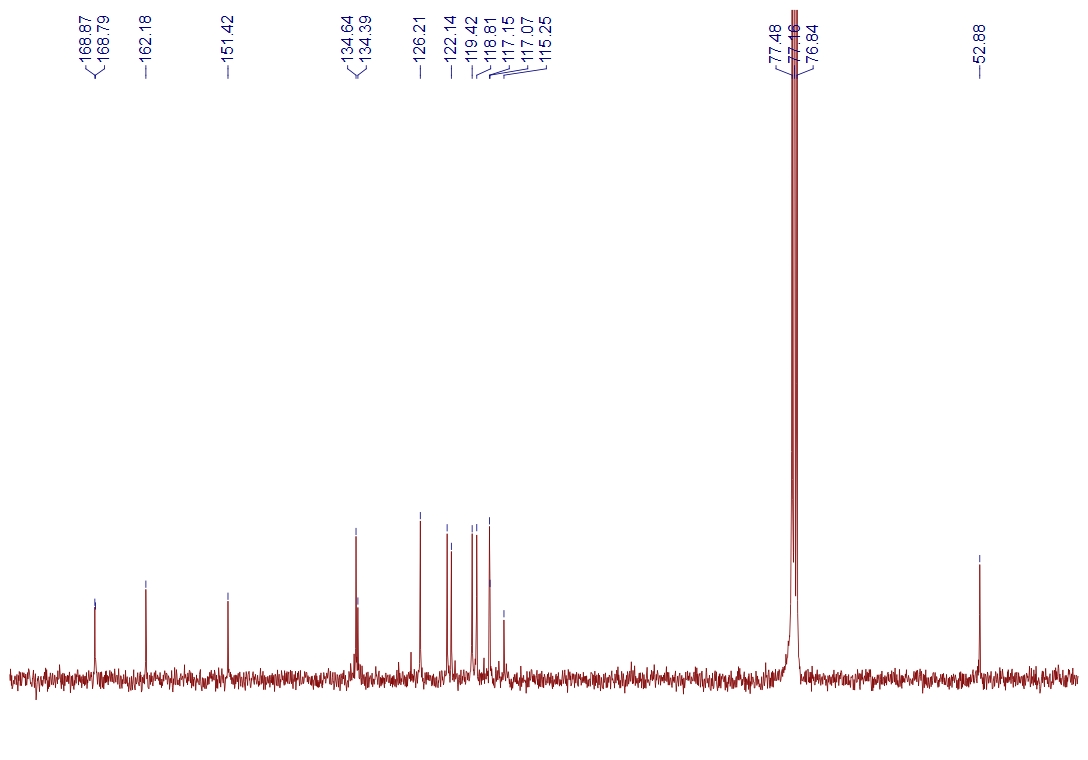


**Fig S9**. ^13^C NMR spectrum of **2** in CDCl_3_.


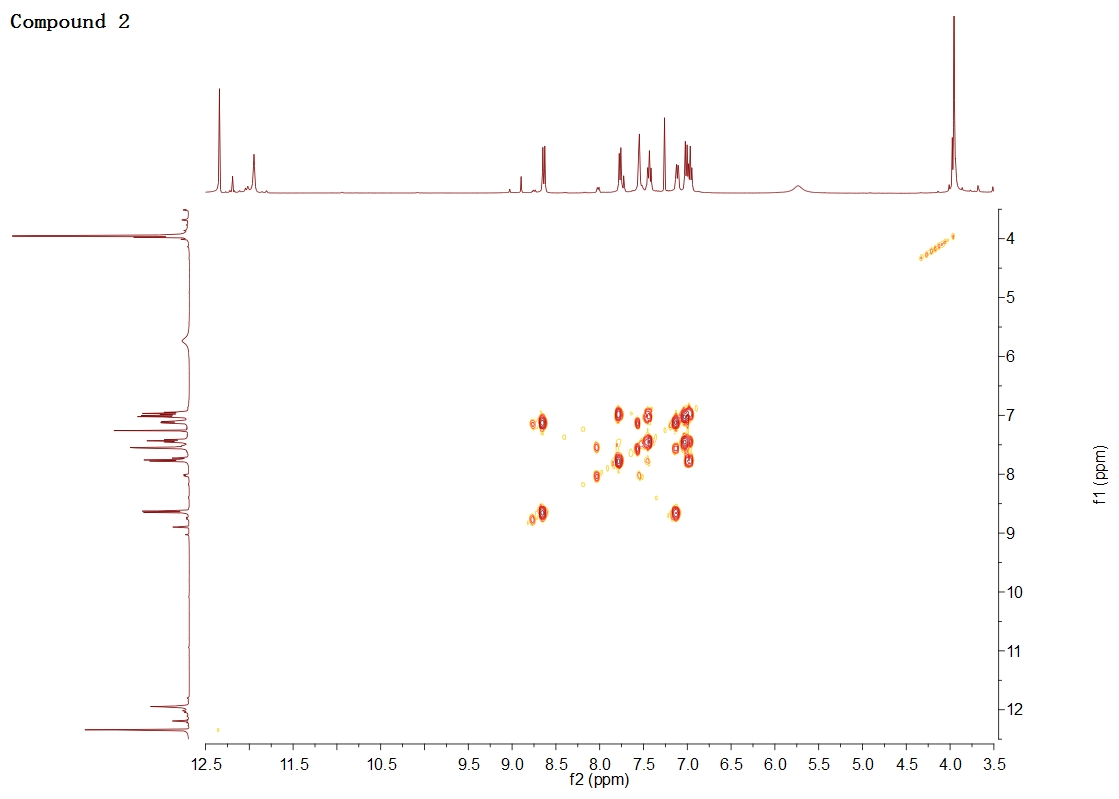


**Fig S10**. ^1^H-^1^H COSY spectrum of **2** in CDCl_3_.

**
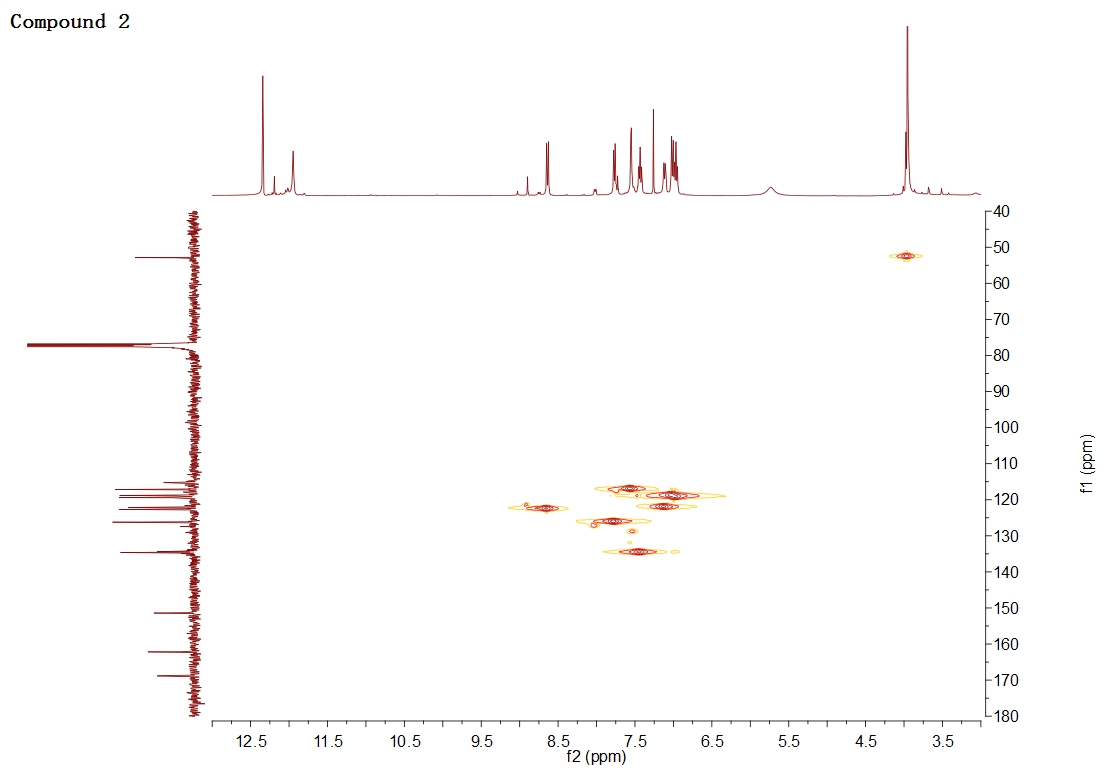
**

**Fig S11**. HSQC spectrum of **2** in CDCl_3_.


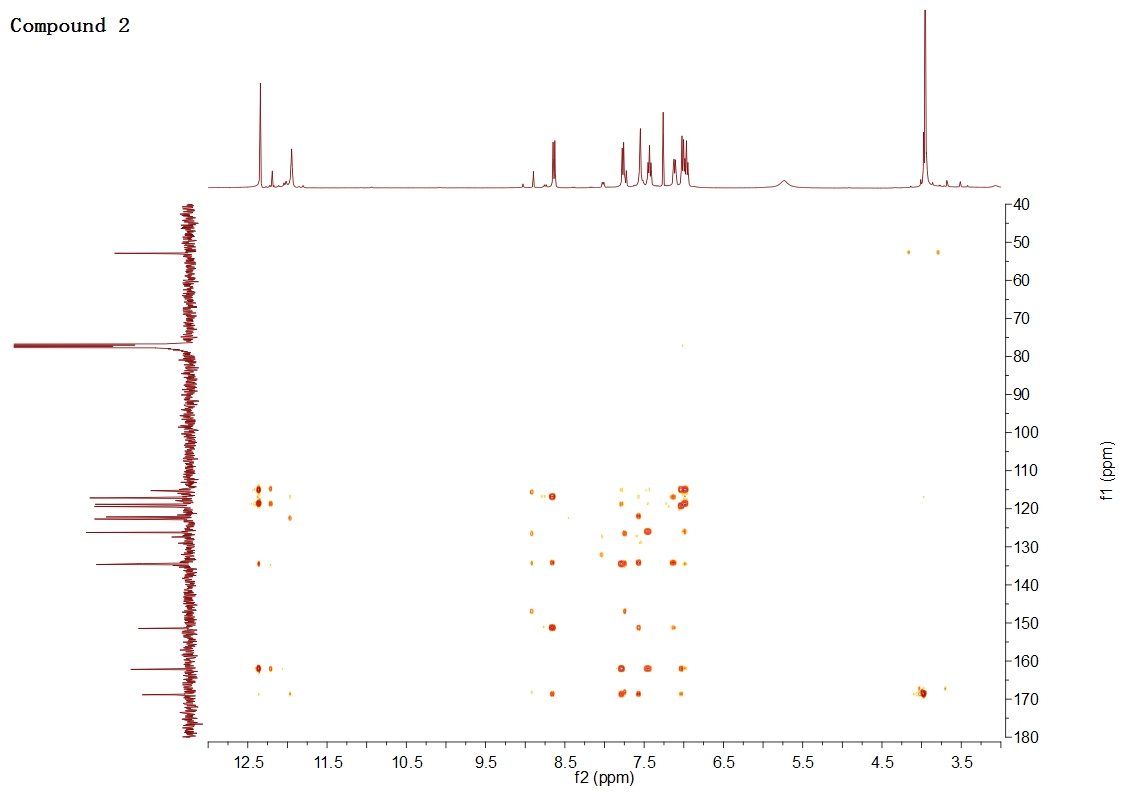


**Fig S12**. HMBC spectrum of **2** in CDCl_3_.

**
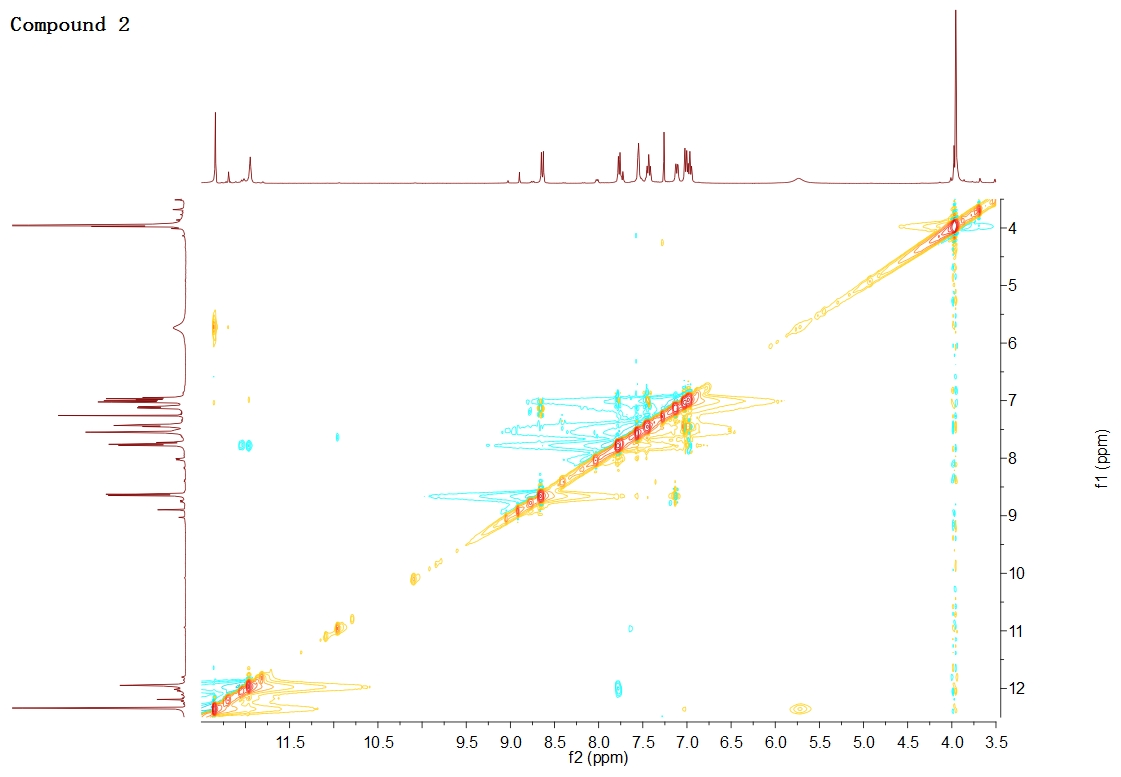
**

**Fig S13**. ROESY spectrum of **2** in CDCl_3_.


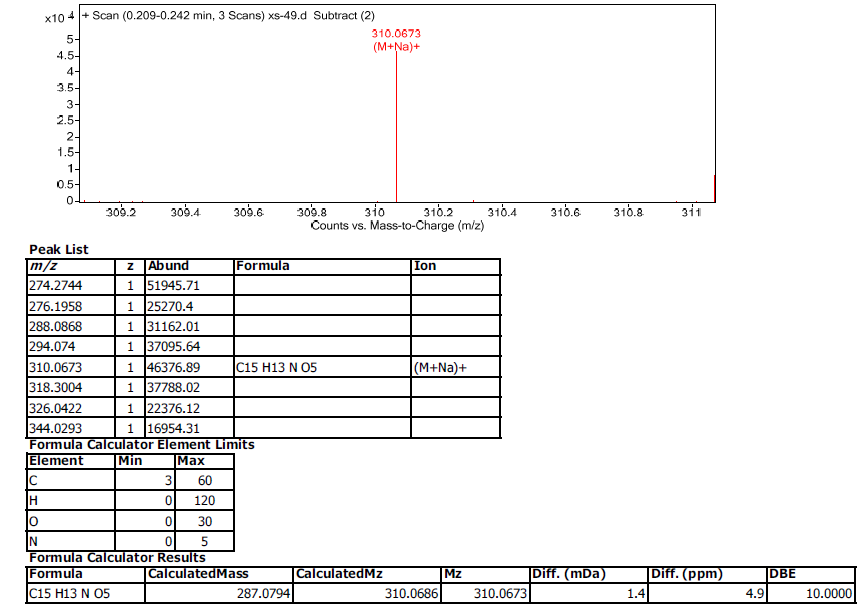


**Fig S14.** HR-ESI-MS spectrum of **2**.
